# Wilson disease, ABCC2 c.3972C > T polymorphism and primary liver cancers: suggestions from a familial cluster

**DOI:** 10.1186/s12881-020-01165-0

**Published:** 2020-11-18

**Authors:** Giovanni Brandi, Alessandro Rizzo, Marzia Deserti, Valeria Relli, Valentina Indio, Sofia Bin, Milena Pariali, Andrea Palloni, Stefania De Lorenzo, Francesco Tovoli, Simona Tavolari

**Affiliations:** 1 Division of Oncology, Azienda Ospedaliero-Universitaria of Bologna, Bologna, Italy; 2 Department of Experimental, Diagnostic and Specialty Medicine, S. Orsola-Malpighi University Hospital, via Massarenti 9, 40138 Bologna, Italy; 3 Center for Applied Biomedical Research, S. Orsola-Malpighi University Hospital, Bologna, Italy; 4 ”Giorgio Prodi” Cancer Research Center, University of Bologna, Bologna, Italy; 5 Department of Medical and Surgical Sciences, S. Orsola-Malpighi University Hospital, Bologna, Italy

**Keywords:** Hepatocellular carcinoma, Intrahepatic Cholangiocarcinoma, ABCC2 c.3972C > T polymorphism, Wilson disease, Risk factors

## Abstract

**Background:**

Polymorphisms in genes modulating xenobiotics metabolism, in particular the ABCC2 c.3972C > T single nucleotide polymorphism (SNP) at exon 28, have been suggested to increase primary liver cancer (PLC) risk. Conversely, the occurrence of PLCs in Wilson disease patients is a rare event, in contrast with the occurrence observed in other chronic liver diseases. Here we report the clinical case of five siblings carrying the ABCC2 c.3972C > T SNP; three of them were affected by Wilson disease and two brothers with Wilson disease also developed PLCs.

**Methods:**

The presence of the ABCC2 c.3972C > T SNP was assessed by Sanger sequencing and the exposure of PLC risk factors by standardized questionnaires.

**Results:**

Notably, PLCs occurred only in the two brothers with the ABCC2 c.3972C > T SNP and Wilson disease who resulted exposed to asbestos and cigarette smoking, but not in the other siblings with the ABCC2 c.3972C > T SNP, alone or in association with Wilson disease, not exposed to these carcinogens and/or to other known risk factors for PLCs.

**Conclusions:**

These findings suggest that ABCC2 c.3972C > T SNP and WD, also in association, may not represent a sufficient condition for PLC development, but that co-occurrence of further host/exogenous risk factors are needed to drive this process, reinforcing the notion that liver carcinogenesis is the result of a complex interplay between environmental and host genetic determinants. Due to the sporadic cases of this study and the paucity of data currently available in literature on this issue, future investigations in a larger population are needed to confirm our findings.

**Supplementary Information:**

The online version contains supplementary material available at 10.1186/s12881-020-01165-0.

## Background

Wilson disease is a rare autosomal recessive genetic disorder linked to a mutation of the copper-transporting P-type ATPase (ATP7B) gene, encoding the ATP7B protein, the main regulator of the hepatic metabolism of copper in the body [1]. As a result, large amounts of copper deposit in the liver and, entering into the systemic circulation, finally accumulate in the brain, kidneys and other vital organs [1]. Clinical presentation can be very heterogeneous but the pathognomonic features of Wilson disease are liver disease and cirrhosis, Kayser–Fleischer rings in Desçemet’s membrane of the cornea, neuropsychiatric symptoms and acute episodes of hemolysis [1]. Despite copper overload is surely involved in the early stage of liver damage, chronic inflammation and cirrhosis, its direct role in liver carcinogensis still remains to be fully clarified.

The development of hepatocellular carcinoma (HCC) and intrahepatic cholangiocarcinoma (iCCA), the two main primary liver cancers (PLCs), is a rare event in Wilson disease patients, in contrast with the occurrence observed in other chronic liver diseases [2]. Conversely, recent studies suggest a potential role of some single nucleotide polymorphisms (SNPs) in increasing PLC susceptibility. Among these, the c.3972C > T genetic variant at exon 28 of the ABCC2 gene has been reported to significantly associate with both HCC and iCCA increased risk [3, 4]. ABCC2 is an ATP-binding cassette (ABC) transporter involved in phase III metabolism of carcinogens and toxins and plays a critical role in cellular defence. In the liver this transporter regulates the pharmacokinetics of many drugs and xenobiotic metabolism by the excretion of conjugates carcinogens into bile [5].

In the present study we report a familiar cluster of five siblings who were found to carry the ABCC2 c.3972C > T genetic variant. Three of them were affected by Wilson disease and two brothers with Wilson disease also developed HCC and/or iCCA; the remaining two siblings are currently without any evidence of Wilson disease and/or PLC development.

## Methods

### Risk factors assessment

The presence of known risk factors for HCC and iCCA in the five siblings was investigated after an accurate anamnesis of their clinical history. Information on cigarette smoking was evaluated by a questionnaire, as previously reported [6]. Subjects smoking ≤100 cigarettes (the equivalent of 5 packs) in their lifetime were classified as never smokers; subjects smoking > 100 cigarettes in their lifetime were classified as current or former smokers, depending on whether they had smoked cigarettes in the past 30 days. Duration of smoking (years) was calculated as follows: present age - age of smoking onset for current smokers; age at quitting cigarettes - age of smoking onset for former smokers. Subjects were also asked about the mean number of cigarettes/day. Pack-years was calculated as follows: number of cigarette packs/day x number of years smoking.

Information on asbestos exposure was assessed according to National Registry of Mesotheliomas (*ReNaM)* questionnaire, as previously reported [7].

### Genomic DNA extraction and sanger sequencing

Genomic DNA was extracted from peripheral blood lymphocytes of the five siblings using the QIAampR DNA Blood Maxi Kit (QIAGEN, Valencia, CA, USA), according to the manufacturer’s protocol. For detection of c.3972C > T SNP at exon 28 in ABCC2 gene (Reference Sequence NM_000392.4), PCR amplification was performed with the following primer sequences: Forward: 5′-CACTGCTACCCTTCTCCTGT-3′; Reverse: 3′-GACCCTTTCCCTCCATCCAA-5′. Amplification conditions were 95 °C for 10 min, 35 cycles at 95 °C for 30s, 60 °C for 30s, 72 °C for 45 s, and finally 72 °C for 7 min. Purified PCR products were then sequenced with Big Dye v1.1 (Life Technologies) according to manufacturer’s instructions and the reaction products analyzed on 3730 DNA Analyzer (Life Technologies, Carlsbad, CA, USA).

## Results

In December 2013 a 56-year-old male patient *presented at our center with a significant weight loss; a contrast enhanced computed tomography (CT) scan revealed* a hepatic lesion measuring 35 × 22 mm in diameter, occupying segment IV. *The patient received genetic* diagnosis of Wilson disease *w*hen he was 43 years old. Blood tests excluded HBV and HCV infection and no evidence of non-alcoholic fatty liver disease/non-alcoholic steatohepatitis was identified by histological examination of hepatic biopsy. The patient reported a history of > 45 pack-year of cigarette smoking and a job as railroader. According to *standardized questionnaires* [6, 7]*, he* resulted an active heavy smoker and moderately exposed to asbestos. The patient underwent wedge resection of segment IV and the histological examination was compatible with iCCA, moderately differentiated (G2). In May 2014, a CT scan demonstrated a new focal lesion in the segment VII and another wedge resection was performed. Histology revealed HCC (G1, R0, N0). He remained without evidence of disease for over 5 years.

The second case refers to the brother, who *received genetic* diagnosis of Wilson disease when he was 36 years old. Fifteen years later, in December 2016, an abdominal ultrasound revealed the presence of four hypoechoic liver lesions occupying segments II, V, VI and VII-VIII, confirmed by a contrast-enhanced ultrasound (CEUS) exam and a CT scan. A biopsy of the VII-VIII segment lesion was performed and histology was consistent with well-differentiated (G1) iCCA. In January 2017, the patient underwent surgery, with no metastatic lymph nodes and radical margins. Post-operative histological examination confirmed the diagnosis of well-differentiated (G1) iCCA. In May 2017, the patient was referred to our centre for the first time and we decided to start a follow-up program; given the time elapsed between surgery and our clinical evaluation, we excluded adjuvant chemotherapy. In January 2018, CEUS, FDG-PET and abdominal MRI detected at least six focal hepatic lesions, suspected for disease recurrence. In a multidisciplinary approach, orthotopic liver transplantation (OLT) was considered only after a conversion treatment strategy using Gemcitabine-Cisplatin schedule. We started the treatment in February 2018 with Gemcitabine-Cisplatin for a total of 4 cycles; in June 2018, the radiological reassessment via CT-PET and MRI revealed partial response, with significant numerical and dimensional decrease of the target lesions. Two months later, the patient underwent OLT; 1 year after OLT, he is without evidence of disease. Similarly to the brother, the patient reported a history of > 50 pack-year of cigarette smoking and worked as railroader, resulting an active heavy smoker and moderately exposed to asbestos, according to *standardized questionnaires* [6, 7]. No other risk factors *linked to iCCA development* [8] emerged from the clinical history.

**Fig. 1:**
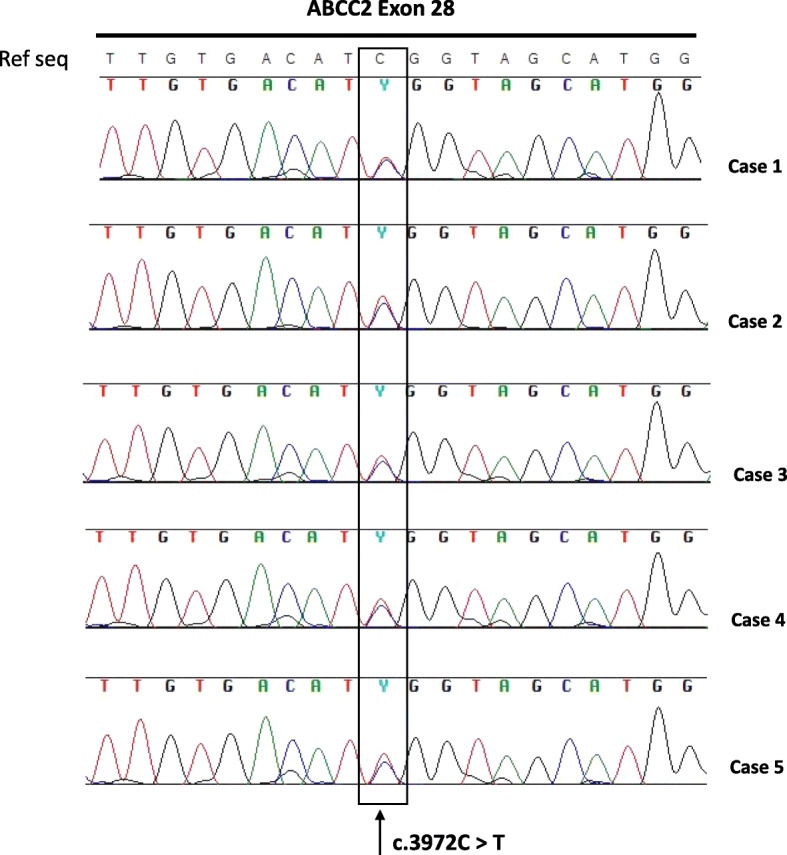
DNA Sanger sequencing from the peripheral blood lymphocytes of the five siblings (Cases 1–5), showing the presence of c.3972C > T SNP at exon 28 of ABCC2 gene in all the samples. SNP: single nucleotide polymorphism; Ref Seq: reference sequence

**Fig. 2:**
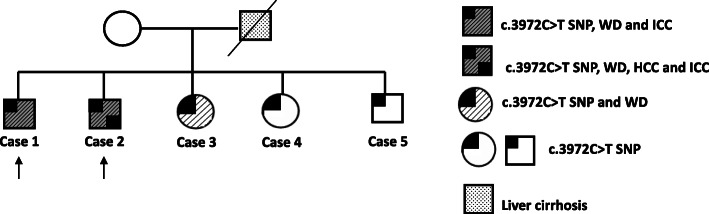
Family pedigree. Type of diseases are shown in the legend. Black arrows show the two brothers with concomitant c.3972C > T SNP at exon 28 of ABCC2 gene, Wilson disease and primary liver malignancies. SNP: single nucleotide polymorphism; WD: Wilson disease; HCC: hepatocellular carcinoma; iCCA: intrahepatic cholangiocarcinoma

Due to the extreme rarity of PLC occurrence in two brothers affected by Wilson disease, we hypothesized that further risk factors may be involved in cancer development. Basing on their history of active heavy smokers and moderate exposure to asbestos, as well as on the reported link between increased PLC risk and the ABCC2 c.3972C > T SNP [3], the presence of this genetic variant involved in xenobiotic metabolism was investigated in the brothers. As shown in Fig. 1 (Cases 1 and 2), they both resulted carriers of the ABCC2 c.3972C > T SNP. Notably, also the other three siblings resulted positive for this SNP (Cases 3, 4 and 5). Among these three siblings, only a sister *received genetic* diagnosis of Wilson disease when she was 31 years old, while the others had no evidence of Wilson disease and/or PLCs (Fig. 2). The father died of liver cirrhosis, although it was not possible to determine if related to Wilson disease or not. *Unfortunately, neither the mother nor other relatives were available for genetic testing and therefore we were unable to assess the segregation of* Wilson disease *and* ABCC2 c.3972C > T *variant in the family.*

## Discussion

In the present study we report the clinical case of five siblings who were found to carry the ABCC2 c.3972C > T SNP; among them, three were affected by Wilson disease and two brothers with Wilson disease also developed PLCs during their life.

The occurrence of PLCs in Wilson disease patients is considered a rare event - especially in iCCA - and the underlying molecular mechanisms have not been yet fully clarified. Sporadic cases of HCC have been described in literature, especially in male subjects, while the occurrence of iCCA is even rarer [9]; moreover, to our knowledge, the development of HCC and iCCA in a patient with Wilson disease has never been reported so far.

A recent multicenter cohort study analyzed 1186 patients affected by Wilson disease; the incidence of PLCs was 0.28 per 1000 person years, with a prevalence of 1.2% [10]. Moreover, the incidence of HCC and iCCA for individuals with Wilson disease have been found to be 0.7 and 0.5%, respectively. These incidences are extremely low if compared to those ones occurring in other bile duct disorders and liver diseases, such as primary sclerosing cholangitis, cirrhosis, viral hepatitis and hepatolithiasis [8]. Currently only sixteen cases of iCCA associated with Wilson disease have been reported in literature, including the two patients of the present study (Supplementary Table 1). Interestingly, although the overall low number of patients precludes the possibility of making strong statements regarding gender differences, a slight prevalence of male subjects has been observed (62.5%, 10/16 cases reported). Previous studies about iCCA in Wilson disease are mainly case reports and case series on a very small number of patients. Sperling et al. reported a patient undergoing liver transplantation for Wilson disease [11], while Saito et al. described a diagnosis of iCCA following a right hepatic lobectomy in a patient with Wilson disease [12]. Similarly, HCC is considered a rare finding in Wilson disease, with approximately forty cases reported in literature. In a recent multicenter Dutch series, after a median follow up of 15 years, only 2 cases of HCC were reported in 130 patients affected by Wilson disease, with an annual HCC risk of 0.14% for cirrhotic subjects and 0.09% for all patients [13]. From a biological point of view, the protective role of copper against cancer development is still debated. Indeed, while an excess of copper is known to induce DNA damage via reactive oxygen species (ROS) generation [14], a protective role of this metal against cancer development has been also reported [15]. Although chronic liver damage is likely the most important risk factor for PLCs onset, the rarity of these tumours in Wilson disease patients suggests that further host/exogenous factors are likely involved in liver carcinogenesis.

In line with this hypothesis, the two brothers developing PLCs shared a moderate exposure to asbestos and a history of heavy active cigarette smoking. Conversely, no risk factors potentially *linked to HCC and iCCA (including exposure to environmental carcinogens such as smoking and asbestos)* emerged from the clinical history of the other three siblings. As to asbestos, a not-negligible increased risk for PLCs, particularly for iCCA, has been recently reported in workers occupationally exposed to this compound [16–18]. For cigarette smoking, some recent meta-analyses reported a moderate association with PLCs risk [19], even if the effective role in the onset of these diseases still remains to be fully clarified. It is worth to underline, however, that in the two brothers developing PLCs, a moderate exposure to asbestos and a history of active cigarette smoking co-occurred with the presence of the ABCC2 c.3972C > T genetic variant. Notably, this variant has been detected with more frequency in iCCA patients compared to healthy population (39.2% vs 26.0%, *p* = 0.022) with an odds ratio = 1.83 (95% CI 1.09–3.08) [3]; similarly, this variant has been significantly associated with increased HCC risk in a study comparing 53 patients and 57 healthy controls [4]. In the liver the ABCC2 transporter is expressed on the membrane of both hepatocytes and cholangiocytes and plays a critical role in xenobiotic metabolism by biliary excretion of toxins and carcinogens [20]; in particular, it regulates the metabolism of the 4-(methylnitrosamino)-1-(3-pyridyl)-1-butatone, one of the most toxic procarcinogens found in tobacco [21]. Although the ABCC2 c.3972C > T SNP identified in the five siblings was a synonymous genetic variant not inducing changes in the amino acid sequence of the protein, it may significantly affect protein function of absorption/excretion of toxins and carcinogens due to aberrant folding, similarly to what reported for a synonymous SNP occurring in the multi-drug resistance 1 gene [22]. It could be therefore hypothesized that in the two brothers with Wilson disease and a history of heavy active smokers and moderate exposure to asbestos, the presence of ABCC2 c.3972C > T genetic variant may have lead to an increased concentration of these carcinogens in the liver tissue over the years, thereby promoting PLC development. Conversely, the lack of PLC development in the three siblings with ABCC2 c.3972C > T SNP alone or in association with Wilson disease, but not exposed to these carcinogens and other known risk factors for PLC, suggest that these two genetic conditions may be not sufficient for PLC development, but that co-occurrence of further host/exogenous risk factors are likely needed to drive this process.

## Conclusions

Overall these findings reinforce the notion that liver carcinogenesis is the result of a complex interplay between environmental and host genetic determinants. However, because of the sporadic cases analyzed in this study and the paucity of data currently available in the literature on this issue, future investigations in a larger population of subjects are needed to confirm our findings.

## Supplementary Information

**Additional file 1:Supplementary Table 1**: Published studies about ICC development in WD patients [23–25].

**Additional file 2:Supplementary file** including ABCC2 gene sequencing raw data.

## Data Availability

All genomic data generated or analysed during this study are available as supplementary file. ABCC2 reference sequence is available at https://www.ncbi.nlm.nih.gov/nuccore/NM_000392.4 web site.

## Abbreviations

HCC: Hepatocellular carcinoma
iCCA: Intrahepatic cholangiocarcinoma
PLC: Primary liver cancer
SNP: Single nucleotide polymorphisms
CT: *Computed tomography*
CEUS: Contrast-enhanced ultrasound
OLT: Orthotopic liver transplantation
