# Supplementary material for: Wilson disease, ABCC2 c.3972C > T polymorphism and primary liver cancers: suggestions from a familial cluster
Source: BMC Med Genet. 2020 Nov 18;21:225. doi: 10.1186/s12881-020-01165-0 (PMC7673086; doi:10.1186/s12881-020-01165-0)
Supplement: Supplementary file 1 — Additional file 1:Supplementary Table 1: Published studies about ICC development in WD patients [23–25]. [file 12881_2020_1165_MOESM1_ESM.doc]

**Supplementary Table 1:** Published studies about ICC development in WD patients.

| **References** | **Sex** | **Age at tumor diagnosis**  **(years)** | **Time from WD diagnosis and tumor detection (years)** | **Therapy for ICC** | **Patient status** |
| --- | --- | --- | --- | --- | --- |
| Present study, Case #1 | M | 56 | 13 | Surgical resection for ICC | Alive after 60 months, NED |
| Present study, Case #2 | M | 60 | 15 | Surgical resection; chemotherapy (GEM/CDDP); liver transplantation | Alive after 30 months, NED |
| Mavilia, 2019 [23] | M | 66 | 66 | Not treatable | DWD after 4 month |
| Nemeth, 2017 [24] | M | 46 | 15 | Not treatable | DWD after 1 month |
| Mukai, 2016 [25] | M | 44 | 20 | Surgical resection | Alive after 44 months, NED |
| Pfeiffenberger, 2015 [10] | F | 70 | 41 | Surgical resection | NA |
| Pfeiffenberger, 2015 [10] | M | 53 | 22 | Orthotopic liver transplantation, chemotherapy (GEM/CDDP) | DWD after 26 months |
| Pfeiffenberger, 2015 [10] | M | 43 | 19 | Chemotherapy (GEM) | DWD after 12 months |
| Pfeiffenberger, 2015 [10] | F | 33 | 6 | Chemotherapy (GEM/CDDP) | NA |
| Pfeiffenberger, 2015 [10] | M | 72 | 41 | Surgical resection | NA |
| Pfeiffenberger, 2015 [10] | M | 56 | 7 | RFA, chemotherapy (GEM/CDDP) | DWD after 12 months |
| Sperling, 2014 [11] | NA | 55 | NA | Liver transplantation for primary tumor (radiation and right hemihepatectomy for recurrence) | Alive after 48 months, NED |
| Saito, 2009 [12] | M | 39 | 28 | Right lobectomy | NA |
| Walshe, 2003 [26] | F | 85 | 22 | NA | NA |
| Walshe, 2003 [26] | F | 40 | 24 | NA | NA |
| Walshe, 2003 [26] | F | 28 | 15 | NA | NA |

WD: Wilson Disease; ICC: intrahepatic cholangiocarcinoma; NA: not available; F: female; M: male; GEM: gemcitabine; CDDP: cisplatin; NED: no evidence of disease; DWD: died with disease.
