# Supplementary figures and images for: Wilson disease, ABCC2 c.3972C > T polymorphism and primary liver cancers: suggestions from a familial cluster
Source: BMC Med Genet. 2020 Nov 18;21:225. doi: 10.1186/s12881-020-01165-0 (PMC7673086; doi:10.1186/s12881-020-01165-0)

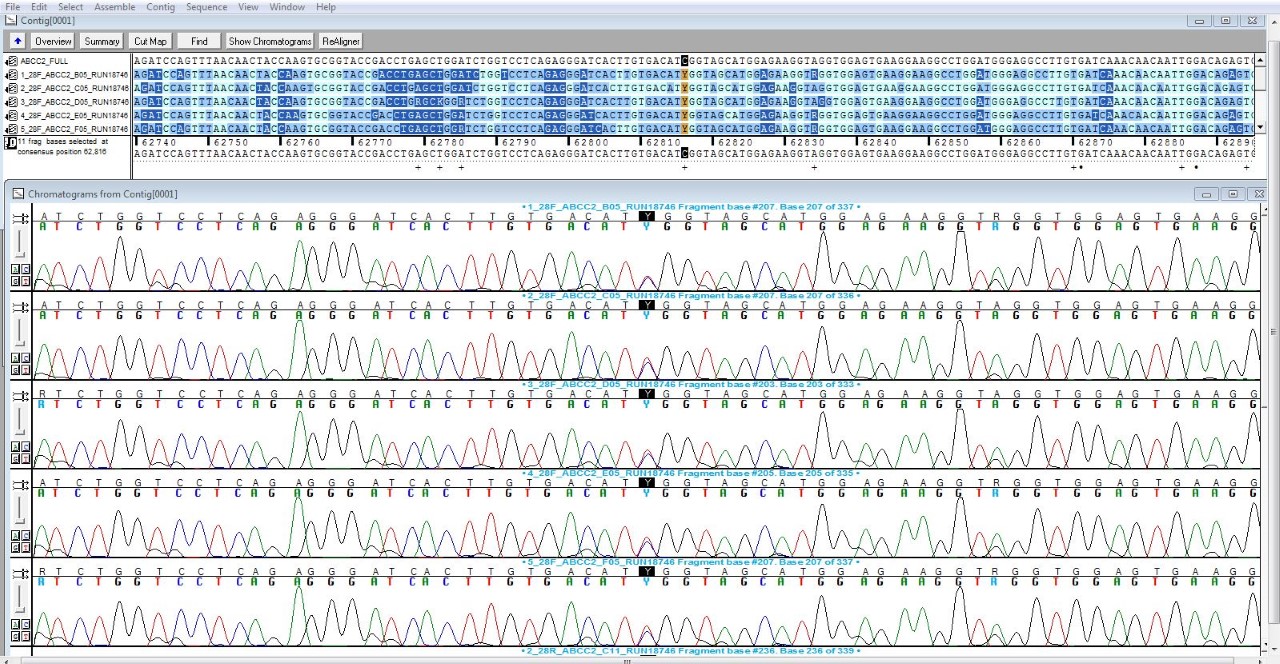

Supplement: Supplementary file 2 — Additional file 2:Supplementary file including ABCC2 gene sequencing raw data. [file 12881_2020_1165_MOESM2_ESM.jpg]
